# Effect of intravenous thrombolysis combined with endovascular treatment on vascular recanalization rate and peak systolic velocity in patients with acute cerebral infarction

**DOI:** 10.12669/pjms.39.5.7573

**Published:** 2023

**Authors:** Hui Zheng, Bo Zheng, Shu Yang, Xin Mou, Xuan Zhang, Huiying Huang, Xiaoping Wu

**Affiliations:** 1 Hui Zheng, Department of Neurology, Chengdu First People’s Hospital, Chengdu 610000, Sichuan Province, P.R. China; 2 Bo Zheng Department of Neurology, Yaan People’s Hospital, Yaan 625000, Sichuan Province, P.R. China; 3 Shu Yang Department of Neurology, Sichuan Academy of Medical Sciences and Sichuan Provincial People’s Hospital, Chengdu 610000, Sichuan Province, P.R. China; 4 Xin Mou, Department of Neurology, Chengdu First People’s Hospital, Chengdu 610000, Sichuan Province, P.R. China; 5 Xuan Zhang, Department of Neurology, Chengdu First People’s Hospital, Chengdu 610000, Sichuan Province, P.R. China; 6 Huiying Huang Department of Neurology, People’s Hospital of Leshan, Leshan 614000, Sichuan Province, P.R. China; 7 Xiaoping Wu, Department of Neurology, Chengdu First People’s Hospital, Chengdu 610000, Sichuan Province, P.R. China

**Keywords:** Acute cerebral infarction, Endovascular treatment, Intravenous thrombolysis, Peak systolic velocity, Vascular recanalization rate

## Abstract

**Objectives:**

To investigate the efficacy of intravenous thrombolysis (IVT) combined with endovascular treatment (EVT) on vascular recanalization rate and peak systolic velocity (PSV) in patients with acute cerebral infarction (ACI).

**Methods:**

A retrospective observational study was conducted from January 2019 to December 2021 in Chengdu First People’s Hospital. The clinical data of 96 patients with ACI were reviewed and the patients were assigned to either the control group (IVT alone, n=54) or the observation group (IVT+EVT, n=42). The vascular recanalization rate, PSV, neurological function, modified Rankin Scale (mRS) score and major adverse cardiovascular events (MACE) were compared between groups.

**Results:**

The vascular recanalization rate and PSV in the observation group were higher than the control group (*P*<0.05). The NIHSS scores of the observation group at 24 hour, one week and one month after treatment were significantly lower than those of the control group (*P*<0.05). The mRS scores of the observation group were significantly lower than the control group after treatment (*P*<0.05), while there was no difference in the incidence of MACE between groups (*P*>0.05).

**Conclusions:**

IVT combined with EVT can improve the vascular recanalization rate and PSV in patients with ACI, which is worthy of promotion in clinical practice.

## INTRODUCTION

Acute cerebral infarction (ACI), or acute ischemic stroke, is one of the leading causes of disability and death worldwide, with 143 million disability-adjusted life-years and 6.55 million mortality in 2019, respectively.^1^ ACI occurs when there is a cerebral artery thrombosis or embolic occlusion.^2^ Treatment of ACI involves dredging of the obstructed blood vessels in time and restoring the oxygen and blood supply to the infarcted area.

Intravenous thrombolysis (IVT) is considered the gold standard treatment for ischemic stroke^3^ and improves neurological function.^4^ However, the recanalization rate of IVT on ACI with large vessel occlusion is low.^5^ In recent years, significant progress has been made in endovascular treatment (EVT), which has greatly increased the recanalization rate and improved the prognosis of ACI patients with large vessel occlusion.^6^ Many studies have investigated the effect of combined IVT with EVT (IVT+EVT) on patients with ACI,^7^-^9^ however, results are conflicting. Our objective was to investigate the effect of IVT+EVT on recanalization rate and peak systolic velocity (PSV) to provide clinical reference for the treatment of patients with ACI.

## METHODS

A retrospective observational study was conducted from January 2019 to December 2021 in Chengdu First People’s Hospital. Clinical data of 96 patients who were diagnosed as ACI was retrospectively reviewed. The patients were assigned to two groups according to the treatment received: control group (IVT only, n=54) and observation group (IVT+EVT, n=42).

### Inclusion Criteria:

- Between the age of 18- 80 years old.
- Patients diagnosed with ACI.^10^
- First onset of ACI.
- Patients whose time from onset of ACI to hospital admission was less than 4.5 hours.
- Blood pressure under 180/100 mmHg.

### Exclusion Criteria:

- Patients with intracranial hemorrhage.
- Patients with contraindications to IVT or EVT.
- Patients complicated with other malignant tumors in heart, liver and kidneys.
- Patients with severe mental disorders.
- Patients who have underwent any surgery within the last three months.
- Patients with incomplete clinical or follow-up data.

### Ethical Approval

The study was approved by the ethics committee of Chengdu First People’s Hospital (No. LL2021022, Date: 2021-02-01), and informed consent was waived due to the retrospective nature of this study.

### Treatment Methods:

### Control group

Patients in the control group were treated with IVT only. Patients were given 0.9mg/kg alteplase (rt-PA, Boehringer Ingelheim Pharma GmbH & Co. KG, Germany) based on body weight, with maximum 90 mg.^11^ First, 10% of the total dose was given as an initial bolus over one minute. Then the remaining 90% dose was infused intravenously over one hour.^11^

### Observation group

Patients in the observation group with arterial occlusion confirmed by magnetic resonance angiography (MRA) or CT angiography were treated with combined IVT and EVT. The IVT method was the same as the control group. After the completion of IVT, digital subtraction angiography (DSA) was performed to identify the occlusive site of the patients. According to the examination results, appropriate EVT interventions including mechanical thrombectomy, balloon dilation and stent placement were selected. The specific surgical procedures have been detailed in previous published literature.^12^ After the operation, the patient was monitored for 24 hours by electrocardiogram. Patients in both groups underwent CT examination 24 hours after IVT. If there was no intracranial hemorrhage, patients were given combination antiplatelet therapy with aspirin (100 mg/d) and clopidogrel (75 mg/d).

### Follow up

Patients were followed up for three months post-treatment. Their neurological assessment was recorded at one month after treatment, and their modified Rankin Scale (mRS) score and major adverse cardiovascular events (MACE) were recorded at three months after treatment.

### Observational Indicators:

### Vascular recanalization rate

The recanalization of occluded blood vessels was examined by transcranial Doppler 24 hours post-treatment, and assessed according to the thrombolysis in cerebral infarction (TICI) grading system.^13^ The TICI grading system is categorized into six levels:^13^ Grade- 0, no perfusion; Grade-1, penetration with minimal perfusion; Grade-2, partial perfusion; Grade-2a, partial perfusion (less than 2/3) of the affected territory; Grade- 2b, complete perfusion of all the affected territory but the perfusion is slower than usual; Grade-3, complete perfusion. In our study, the TICI grading = 0, ≥2a and =3 was considered as vascular blocked, partial recanalization and complete recanalization, respectively.

### Peak systolic velocity (PSV) of the vertebral artery

Internal carotid artery and common carotid artery were measured by transcranial Doppler ultrasound in both groups before and 24 hours post-treatment.

### Neurological function

The National Institutes of Health Stroke Scale (NIHSS)^14^,^15^ was used to assess the neurological function of patients in both groups before treatment, 24 hours post-treatment, one-week post-treatment and one-month post-treatment. The score range was 0 - 42, with higher scores representing severer neurological deficits. It mRS is a 6-point disability scale with scores ranging between 0-5. A three months mRS score of ≤ 2 was considered a good outcome, while a score of 3-5 was considered a poor outcome.^16^

### Major adverse cardiovascular events (MACE)

It includes death, intracranial hemorrhage, transient ischemic attack and cerebral infarction in new territory were recorded in the two groups three months post-treatment.

### Statistical Analysis

All data were analyzed using SPSS 23.0 (IBM, USA). Normally distributed and homogeneity of variance variables were described as mean ± SD and differences between the two groups were compared using Student’s t-test. Not normally distributed variables were described as median (IQR) and differences between the two groups were compared using Mann-Whitney U-test. Paired-sample t-test was used to compare before and after treatment within a group. Repeated measures ANOVA was conducted for comparison of multiple time points. Qualitative variables were described as frequency and percentage (n, %), and differences between the two groups were compared by Chi-square test. *P*<0.05 indicated a statistically significant difference.

## RESULTS

The control group included 35 males and 19 females aged 58-78 years (66.80±4.56 years) and the observation group included 26 males and 16 females aged 55-79 years (66.36 ± 6.59 years). There was no difference in baseline data between groups (*P* > 0.05; Table-I). The vascular recanalization rate in the observation group and the control group post-treatment were 85.7% and 61.1%, respectively. The vascular recanalization rate in the observation group was higher than that in the control group (*P*<0.05; Table-II).

**Table-I:** Baseline data of the participants.

Variables	Observation group (n=42)	Control group (n=54)	t/	P
Gender, male, n(%)	26(61.9)	35(64.8)	0.086	0.832^a^
Age, years, mean ± SD	66.36±6.59	66.80±4.56	-0.385	0.713^b^
BMI, kg/m^2^, median (IQR)	25.50(23.50-28.18)	25.50(21.48-27.60)	-0.821	0.411^c^
Hypertension, n(%)	21(50.0)	30(55.6)	0.293	0.681^a^
Diabetes mellitus, n(%)	12(28.6)	15(27.8)	0.007	1.000^a^
Hyperlipidemia, n(%)	8(19.0)	12(20.8)	0.144	0.803^a^

BMI, body mass index.

a , Chi-square test;

b , Student’s *t*-test;

c , Mann-Whitney U-test.

**Table-II:** Vascular recanalization post-treatment [n(%)]

Variables	Observation group (n=42)	Control group (n=54)		*P*
Complete recanalization	23	15	9.472	0.009
Partial recanalization	13	18		
Blocked	6	21		
Total recanalization rate	85.7%	61.1%		

There were no significant differences in PSV of vertebral artery, internal carotid artery and common carotid artery between groups before treatment (*P*>0.05). Post-treatment, the PSV of the arteries in each group was higher than that before treatment (*P*<0.001). The PSV of the arteries in the observation group post-treatment were significantly higher than those in the control group (*P*<0.001; Table-III).

**Table-III:** Comparison of PSV between groups before and 24 hours post-treatment (cm/s).

Variables	Observation group (n=42)	Control group (n=54)	t/Z	P
***Vertebral artery***				
Before treatment	36.10(34.30-38.23)	35.40(33.05-38.40)	-0.739	0.460^a^
Post-treatment	48.14±3.23*	43±3.28*	6.893	<0.001^b^
***Internal carotid artery***				
Before treatment	45.55(42.38-48.85)	44.10(41.10-47.33)	-1.518	0.129^a^
Post-treatment	55.85±4.17*	49.37±4.04*	7.689	<0.001^b^
***Common carotid artery***				
Before treatment	42.10(41.80-43.13)	42.20(42.00-42.53)	-0.709	0.478^a^
Post-treatment	53.45±3.72*	48.68±3.67*	6.267	<0.001^b^

Data are presented as median (IQR) or mean ± SD.

a , Mann-Whitney U-test;

b , Student’s t-test. Compared within the same group before treatment,

* P < 0.001.

There were no significant differences in NIHSS scores between groups before treatment (*P*>0.05). The NIHSS scores of the observation group at 24 hours, one week and one month post-treatment were significantly lower than the control group (*P*<0.001). The NIHSS scores in each group at these three time points were significantly lower than those before treatment within each group (*P*<0.001). When compared within the same group, the NIHSS scores at one week and one month post-treatment were significantly lower than those at 24 hours and one week post-treatment, respectively (*P*<0.001; Table-IV). The mRS scores of the observation group were significantly lower than the control group after three months of follow-up (*P*<0.05), but there was no difference in the incidence of MACE between the two groups (*P*>0.05; Table-V).

**Table-IV:** Comparison of NIHSS scores between groups (mean ± SD).

Variables	Observation group (n=42)	Control group (n=54)	t	P
Before treatment	18.33±3.00	18.20±3.46	0.193	0.847
24h post-treatment	13.38±1.70^a^	15.93±2.92^a^	-5.350	<0.001
1-week post-treatment	10.00±1.55^a,b^	11.95±2.25^a,b^	-4.784	<0.001
1-month post-treatment	7.88±1.63^a,b,c^	9.69±1.96^a,b,c^	-4.813	<0.001
F	211.811	96.237		
P	<0.001	<0.001		

a P < 0.001, within the same group before treatment; ^b^P < 0.001, within the same group 24h post-treatment; ^c^P < 0.001, within the same group 1-week post-treatment.

**Table-V:** Comparison of mRS and MACE between groups after three months of follow-up.

Variables	Observation group (n=42)	Control group (n=54)		P
***mRS, score, median (IQR)***				
Before treatment	4.00(3.00-5.00)	4.00(3.00-4.25)	-0.054	0.957^a^
Post-treatment	2.00(2.00-3.00)	3.00(2.00-3.00)	-2.420	0.016^a,*^
***MACE, n(%)***				
Intracranial hemorrhage	2(4.8)	6(11.1)		
Infarction in new territory	0(0.0)	2(3.7)		
Transient ischemic attack	1(2.4)	3(5.6)		
Death	0(0.0)	0(0.0)		
Total	3(7.1)	11(20.4)	3.318	0.069^b^

a , Mann-Whitney U-test;

b , Chi-square test. *P < 0.05.

## DISCUSSION

The results presented here show that the combination of IVT and EVT could significantly improve the vascular recanalization rate, neurological function, and PSV, in addition to reducing the incidence of MACE in patients with ACI.

Several studies have reported the effect of IVT+EVT in patients with ACI on the vascular recanalization rate and neurological function compared with patients treated with IVT or EVT alone.^7^-^9^,^17^-^20^ Some studies have found that patients with ACI who received IVT+EVT had better recanalization rates.^8^,^12^,^17^,^20^ A shorter time from symptom onset to recanalization was associated with better clinical outcomes and neurological function in patients with ACI.^12^ Further evidence shows that EVT following IVT may improve the vascular recanalization rate, subsequently improving survival and recovery in patients with acute ischemic stroke.^20^ However, application of IVT+EVT may lead to higher hospital costs without improving the vascular recanalization or functional outcomes.^7^

Our results confirm those in the literature, that patients receiving IVT+EVT had higher vascular recanalization rates and improved neurological function than those who received IVT alone. It is possible that the thrombolytic agent Alteplase promotes the conversion of plasminogen to plasmin, thereby dissolving the thrombus and unblocking the blood vessels. Also, the application of IVT before EVT can help to reduce the thrombus load in large vessels, improving the stability of stent and thrombus riveting. This combination may also help to dissolve secondary thrombus in collateral circulatory pathways slowing down the time of infarction and thus improving neurological function after thrombectomy. EVT can be directly applied to the vascular obstruction site which can shorten the vascular recanalization time.

In the present study, mRS was used to assess the neurological function recovery of the patients and we found that the mRS scores were lower in patients received the IVT+EVT approach, which is consistent with the findings by Huo et al^8^ and Zhu et al^12^. In addition, our findings on the incidence of MACE after treatment support the study by Yang et al^21^ that there was no significant difference in incidence rate of MACE in patients received EVT alone or IVT+EVT. It is suggested that IVT+EVT is as safe as IVT alone and it does not increase the risk of MACE.

Cerebral hemodynamic indexes can reflect the state of cerebral blood vessels and cerebral blood flow velocity, and PSV is a predictor of stroke.^22^ There is limited data on the effect of IVT+EVT on PSV in patients with ACI. In our study, the PSV of patients receiving IVT+EVT was higher than patients receiving IVT alone, suggesting that IVT+EVT may improve cerebral blood flow perfusion in patients with ACI. As IVT+EVT can significantly shorten the vascular recanalization time, this may improve cerebral blood perfusion and avoid tissue hypoperfusion caused by vascular stenosis. Moreover, it can avoid plaque hemorrhage and thrombosis caused by the rupture of fibrous cap of unstable plaque.

### Limitations of the study

The sample size of the study was small, which may limit the generalization of the findings. Future studies with larger sample size should be conducted to further verify our observations. Moreover, it is a retrospective study with limited data, hence it is necessary to carry out randomized controlled trials to further confirm our findings. The follow-up period of the study was only three months, which could be further increased in future research.

## CONCLUSION

The combination of IVT+EVT results in improved vascular recanalization rate, neurological function and PSV compared to IVT or EVT alone in patients with ACI.

### Authors’ contributions:

**HZ:** Conceived and designed the study.

**BZ, SY, XM, XZ, HH and XW:** Collected the data and performed the analysis.

**HZ:** Was involved in the writing of the manuscript and is responsible for the integrity of the study.

All authors have read and approved the final manuscript.
